# Bioinformatics Tools for the Analysis of Active Compounds Identified in Ranunculaceae Species

**DOI:** 10.3390/ph16060842

**Published:** 2023-06-05

**Authors:** Cătălina Mareş, Ana-Maria Udrea, Nicoleta Anca Şuţan, Speranţa Avram

**Affiliations:** 1 Department of Anatomy, Animal Physiology and Biophysics, University of Bucharest, 91-95 Splaiul Independentei, 050095 Bucharest, Romania; catalina.sogor@bio.unibuc.ro (C.M.); speranta.avram@gmail.com (S.A.); 2 Laser Department, National Institute for Laser, Plasma and Radiation Physics, Atomistilor 409, 077125 Magurele, Romania; ana.udrea@inflpr.ro; 3 Research Institute of the University of Bucharest-ICUB, University of Bucharest, 91-95 Splaiul Independentei, 050095 Bucharest, Romania; 4 Department of Natural Sciences, University of Piteşti, 1 Targul din Vale Str., 110040 Pitesti, Romania

**Keywords:** Ranunculaceae, alkaloids, pharmacokinetics, pharmacogenomics, pharmacodynamics, carbonic anhydrases, bioinformatics

## Abstract

The chemical compounds from extracts of three Ranunculaceae species, *Aconitum toxicum* Rchb., *Anemone nemorosa* L. and *Helleborus odorus* Waldst. & Kit. ex Willd., respectively, were isolated using the HPLC purification technique and analyzed from a bioinformatics point of view. The classes of compounds identified based on the proportion in the rhizomes/leaves/flowers used for microwave-assisted extraction and ultrasound-assisted extraction were alkaloids and phenols. Here, the quantifying of pharmacokinetics, pharmacogenomics and pharmacodynamics helps us to identify the actual biologically active compounds. Our results showed that (i) pharmacokinetically, the compounds show good absorption at the intestinal level and high permeability at the level of the central nervous system for alkaloids; (ii) regarding pharmacogenomics, alkaloids can influence tumor sensitivity and the effectiveness of some treatments; (iii) and pharmacodynamically, the compounds of these Ranunculaceae species bind to carbonic anhydrase and aldose reductase. The results obtained showed a high affinity of the compounds in the binding solution at the level of carbonic anhydrases. Carbonic anhydrase inhibitors extracted from natural sources can represent the path to new drugs useful both in the treatment of glaucoma, but also of some renal, neurological and even neoplastic diseases. The identification of natural compounds with the role of inhibitors can have a role in different types of pathologies, both associated with studied and known receptors such as carbonic anhydrase and aldose reductase, as well as new pathologies not yet addressed.

## 1. Introduction

### 1.1. Overview of Ranunculaceae Active Compounds

Interest in Ranunculaceae species, the perennial plant that presents bioactive chemical compounds, has been raised due to the toxic properties that species possess [1]. Herbal medicine has a long history, and lately, it is becoming more popular globally [2,3]. Even if the source of these medicinal compounds is natural, this does not mean that it is safe and has fewer adverse effects [4]. For example, ingestion of aconitine can cause severe cardiotoxicity manifested by ventricular arrhythmia [5].

*Aconitum*, *Helleborus* and *Anemone* are genera of the Ranunculaceae family, comprising valuable species with pharmacological use, such as aconite roots, which have the highest concentration of aconitine (AC). Symptoms due to accidental ingestion of a large amount of the substance are neurological, gastrointestinal and cardiovascular symptoms [6].

The pharmacological effects of AC that have been identified are antitumor, anti-inflammatory, analgesic and local anesthetic effects [7]. These results make AC a potential therapeutic agent for diseases such as cancer, rheumatoid arthritis and chronic pain [8]. In Table 1, we summarize the main study about the pharmacological effects of AC. 

The high toxicity of this compound has determined numerous cases of poisoning, whereby the oral administration of AC can result in mild intoxication to cardiac arrest and death depending on the dose [9]. AC toxicity has become an acute problem, and that is why we are trying to obtain the maximum therapeutic effects at the lowest possible doses of the compound [10].

**Figure 1:**
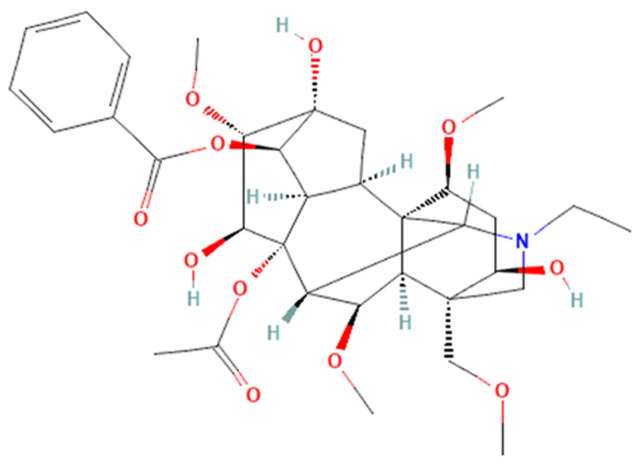
Chemical structure in 2D format of AC (PubChem CID 245005) [16].

The pharmacological mechanisms and pharmacokinetic properties of AC have not been fully understood, but the evidence of its utility in different pathologies could help in the management of different diseases [1]. The pharmacological effects of AC, but also of similar alkaloids such as hypaconitine and mesaconitine, are given by the chemical skeleton of the compounds. In Figure 1, the chemical structure of AC can be identified, with the skeleton being found in C19-diterpenoid alkaloids [11].

Countless studies on tissues of rats and mice show tissue degeneration and necrosis, especially the heart and brain, following the administration of the solution obtained from the decoction of *Aconitum* sp. The data were evaluated according to the dose applied and the incubation time [12,13,14,15].

Natural compounds can be useful in the treatment of central nervous system diseases such as depression, anxiety and inflammatory diseases. Quercetin is a flavonoid with antidepressant activity observed in mice [17]. The anxiolytic and antidepressant effect [17] of quercetin regulates cholinergic and serotoninergic functions [18].

Studies have shown the importance of polyphenols in minimizing neurodegenerative effects [19]. The study by Zhao et al. showed the importance of the therapy of the combination of quercetin and malvidin for the protection of neurons against cognitive dysfunction due to sleep deprivation. Sleep deprivation of mice showed a decrease in memory consolidation capacity, but the mixture of polyphenols improved the negative effects on the studied organisms [20].

Polyphenols are natural compounds with biological effects aimed at their antioxidant effect, improving oxidative stress by activating antioxidant enzymes and inhibiting reactive oxygen species [21]. The relevance of polyphenolic compounds catechin, epicatechin [22], caffeic acid, coumaric acid, chlorogenic acid [23], delphinidin is given by the applicability of these natural compounds in complex diseases such as cardiovascular diseases, neurodegenerative diseases, obesity and cancer [24].

Genistein and gallic acid are part of the family of polyphenols with the role of inhibiting the binding of the nuclear factor of activated B cells (NF-kB) by the target DNA. Polyphenols have been reported in the literature as strong inhibitors of NF-kB, thus being useful in the treatment of various types of dementia, including dementia of the Alzheimer type or Parkinson’s disease [25,26]. Natural compounds such as genistein and gallic acid inhibit the transmission of pro-inflammatory cytokines, neuroinflammation and oxidative stress, which are considered causes of neuronal loss [27].

Syringic acid has a neuroprotective effect, due to the antioxidant, anti-inflammatory and antidepressant properties that polyphenols have [28].

Naringenin and naringin are citrus flavonoids with antioxidant, anti-proliferative and anti-inflammatory activities, thus representing a potential therapeutic effect in preventing and alleviating the symptoms of neurological disorders [29].

Rutin is a powerful antioxidant involved in Alzheimer’s disease and other neurodegenerative pathologies. The pharmacological applications of this compound are mainly due to the antioxidant and anti-inflammatory activity that this compound possesses [17]. The action mechanisms through which rutin can act are conducted via eliminating and inhibiting the production of reactive oxygen species [30].

Magnoflorine is fat soluble and can cross the blood–brain barrier, thus manifesting the antidepressant effect. In vivo studies on model animals have shown that magnoflorine can increase the expression of antioxidant proteins. Magnoflorine is an alkaloid with the function of protecting the cardiovascular system and regulating the immune system, and it has very good antioxidant properties [31]. The medical properties of magnoflorine suggest its antidepressant effect [32].

Hyperoside can improve memory and learning in mice by potentiating synapses and ameliorating memory disorders induced by neurodegenerative diseases [33]. It was also observed, following long-term treatment with hyperoside in mice, the reduction in beta-amyloid plaque and tau protein phosphorylation, the attenuation of neuroinflammation and stress oxidative, thus being a potential drug for Alzheimer’s disease [34].

**Figure 2:**
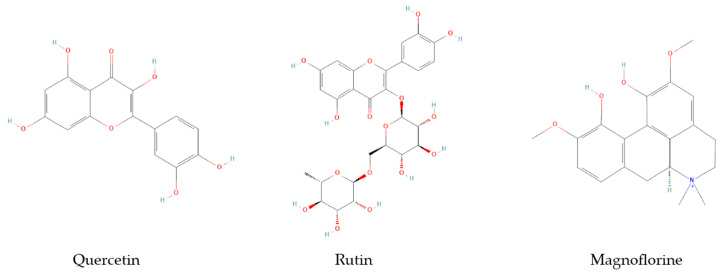
The chemical structures of the compounds that showed the most important antioxidant activity, quercetin (PubChem CID5280343), rutin (PubChem CID5280805), magnoflorine (PubChem CID73337) [35].

The chemical structures of the compounds that showed the most important antioxidant activity can be visualized in Figure 2.

The computational approach in biology and medicine plays an essential role both in the actual diagnosis of diseases and in the development of medicines based on computational methods. The steric and electronic molecular descriptors, the number of atoms and the type of chemical bonds represent essential information in studying the biological activity of compounds based on their chemical structure [36].

The purpose of the current study is to characterize from a bioinformatic point of view the compounds identified from an extract of *Aconitum* sp. and to try to identify those compounds that have a truly biologically active role. 

Genistein has proven to be useful both in the case of diabetes and the complications caused by this disease [37] and also in other disorders such as cataracts, cystic fibrosis, and non-alcoholic fatty liver disease. This compound shows high binding affinity to aldose reductase and inhibits its activity [38].

**Table 1:** Pharmacological effects and mechanism of action of different organisms/cell type determinate by AC.

Pharmacological Effects	Cell Lines	Mechanism of Action	Reference
Anti-inflammatory activity	Mouse leukemic monocyte/macrophage cell line RAW264.7	Suppressing NF-kB and NFATc1 activation and DC-STAMP expression	[39]
Anti-rheumatic activities	Rheumatoid arthritis HFLS-RA fibroblast-like synoviocytes		[40]
Analgesia activity	Male wildtype FVB mice and Male Mdr1a−/− FVB mice	Mdr1a deficiency	[41]
	the rat chronic constriction injury of an infraorbital nerve model.	N-methyl-D-aspartate receptor	[42]
	Mice pain models caused by hot plate, acetic acid, formalin, and CFA	-	[14]
Anti-cancer activity	Pancreatic cancer cell lines Miacapa-2 and PANC-1	Suppressing cancer cell growth and increasing cell apoptosis	[43]
	Human cervical carcinoma HeLa cells	Upregulating mRNA expression levels of eIF2α, ATF4, IRE1, XBP1, ATF6, PERK	[44]
	Human breast cancer cell line MDA-MB-231BO	Inhibiting cancer cell invasion by an alteration of the TGF-β/Smad signaling pathway and down-regulating of NF-κB and RANK expressions.	[45]
	Human OVCA A2780 cell line	Adjusting ERβ-mediated apoptosis, DNA damage and migration	[8]

For a better prediction of the mechanisms, we selected the compounds that showed satisfactory results and performed molecular docking. Molecular docking is a quick method for predicting the interaction of a specific target and a ligand [46,47]. In this study, we will predict the lowest estimated free energy of binding (LEFEB) between quercetin, caffeic acid, chlorogenic acid, coumaric acid, hyperoside, and rutin when they interact with aldose reductase (AKR1B1).

In the current study, the bioinformatics testing of the compounds obtained from the HPLC extraction is conducted with the aim of identifying the antioxidant and anti-inflammatory effect of the compounds. The study wants to identify the most useful compounds for the treatment of anxiety, depression, and neurodegenerative diseases.

### 1.2. The Choice of Natural Compounds

Our study followed the identification of the compounds obtained from microwave and ultrasound extraction of rhizomes, leaves, and/or flowers of *Aconitum toxicum* Rchb., *Anemone nemorosa* L., and *Helleborus odorus* Waldst. & Kit. ex Willd. and the bioinformatics testing of each of them to highlight their role in the extracts [48].

In the present study, we performed an in silico analysis of the compounds identified by HPLC from hydroalcoholic extracts of *A. toxicum*, *A. nemorosa*, and *H. odorus*. The twenty-one compounds identified in the extracts were aconitine, hypaconitine, mesaconitine, magnoflorine, gallic acid, catechin, caffeic acid, ferulic acid, chlorogenic acid, epicatechin, delphinidin, coumaric acid, daidzein, hyperoside, rutin, naringin, malvidin, quercetin, naringenin, genistein, and syringic acid. 

Starting from the literature on natural compounds with effects on the central nervous system, we focus our attention on compounds suitable for the analysis of pathologies in the psychiatric spectrum. Thus, the predicted molecular targets of the studied compounds are generally statistically significant for nervous system pathologies [49].

The study by Avram et al. provides essential information about the molecular descriptors and QSAR equations useful in inducing the antidepressant activity of the different compounds [50].

### 1.3. Molecular Targets Obtain Using Prediction Target Database

The nervous system presents a particular complexity, but with the help of bioinformatics techniques, it is possible to predict the activity of certain compounds for the treatment of anxiety, depression, and even some neurodegenerative diseases [50,51]. Pharmaceutical descriptors (electrostatic, hydrophobic, hydrogen donor/acceptor bond, etc.) can predict the biological activity of the compounds and their effect in the body [50].

The main molecular targets obtained with the help of prediction databases are from the family of aldose reductase and carbonic anhydrase enzymes.

Carbonic anhydrases (CA) are enzymes (16 isoforms) with important roles in the optimal functioning of the body. Their pharmacological inhibition has been reported as a treatment for many diseases (glaucoma, neuropathic pain, tumors) [52]. CA catalyzes the transition from carbon dioxide to bicarbonate and protons [53]. CA inhibition has good prospects for understanding the protein–drug interaction mechanism and designing useful pharmacological agents [54]. CA are metalloenzymes that become active when the pH is basic, the active locus of the enzyme being the hydrophobic pocket in which the zinc ion is found, which is crucial for the catalytic reaction, thus regulating the concentration of CO2 in the test. In mammals, CA functions are represented by respiration, pH regulation, electrolyte secretion, and metabolic processes dependent on HCO3 [55,56].

CA II is an isoform that is identified in the brain and is expressed in neurons, oligodendrocytes, and the choroid plexus [57], and isoform VII is found in the hippocampus and cortex [58]. The scientific literature indicates the involvement of these enzymes in neuropathological processes [56]. CA inhibitors can function as anticonvulsants in animal models of epilepsy and in diagnosed patients. Current studies approach new structures that can inhibit different isoforms of anhydrases to minimize the side effects on patients of existing inhibitors [59].

Aldose reductase is a cytosolic enzyme that belongs to the aldose-keto-reductase family and is found in most mammalian cells. Although the roles of this enzyme in the polyol pathway, the one that transforms glucose into sorbitol, are well known, recent studies suggest the involvement of this enzyme in detoxification processes under conditions of oxidative stress. This enzyme is mainly involved in the complications of diabetes such as retinopathy, cataracts, nephropathy, and neuropathy [60]. Among the useful compounds that inhibit aldose reductase are polyphenolic compounds such as curcumin, quercetin, and kaempferol. These natural compounds possess a strong inhibitory effect on enzymes both in in vitro and in vivo studies [61]. 

Neurodegeneration caused by Alzheimer’s disease is achieved by the precipitation of beta-amyloid in the brain, which produces inflammation [62]. A study by Huang et al. showed in a cell culture of neurons and microglia that inhibition of aldose reductase with sorbinil as a therapeutic agent prevented cell migration and phagocytosis. Thus, the neuronal death in the culture was mitigated, and it was proven that the inhibition of aldose reductase is effective in the neuronal degeneration induced by beta-amyloid [63].

These results are useful for us in the search for new aldose reductase inhibitors that may also have a useful role in the case of Alzheimer’s disease.

## 2. Results

### 2.1. Drug-Likeness, Pharmacokinetics, and Pharmacogenomics Profiles of Compounds

**Figure 3:**
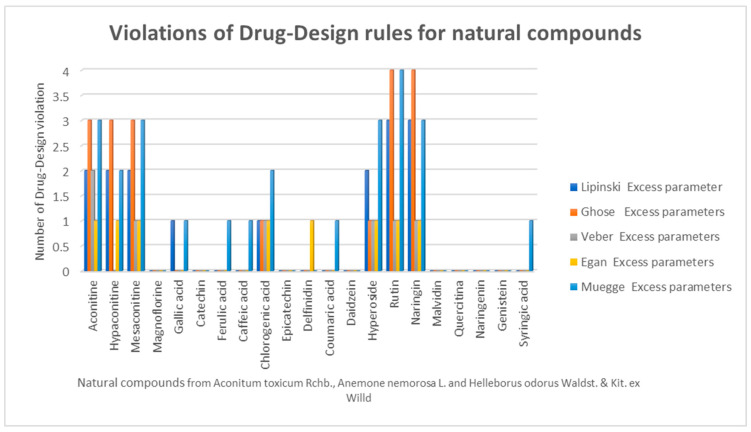
Compliance with the drug-like rules—the Lipinski, Ghose, Veber, Egan, and Muegge rules.

To evaluate the character of a possible drug, the compounds were processed in the Expasy database [64] and tested to comply with the rules of medical chemistry—the Lipinski [65], Veber [66], Egan [67], and Muegge [68] rules. Violations of drug design rules for the analyzed compounds are in Figure 3.

For the mentioned compounds, the physico-chemical properties are calculated, such as molecular weight, hydrophobicity, the share of hydrogen bond donor/acceptor atoms, the share of the number of rotatable bonds, polar molecular surface, and so on. Chemical compounds for which excess is identified are considered not to meet the drug-like condition [50,68].

Through the bioinformatics calculation of the drug-like character, we found that the following compounds respect the drug-like profile: magnoflorine, gallic acid, catechin, ferulic acid, caffeic acid, chlorogenic acid, epicatechin, delphinidin, coumaric acid, daidzein, malvidin, quercetin, naringenin, genistein, and syringic acid. 

**Figure 4:**
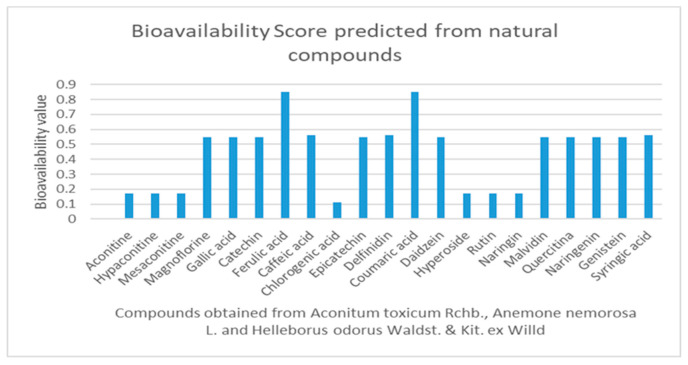
Bioavailability score values for natural compounds obtained from *Aconitum toxicum* Rchb., *Anemone nemorosa* L., and *Helleborus odorus* Waldst. & Kit. ex Willd.

Bioavailability is a very important indicator in the absorption of drugs. A high bioavailability means that the compound will reach the systemic circulation more easily when administered orally. A higher bioavailability means that more nutrients are absorbed by the conventional method. Ferulic acid and coumaric acid show the best availability of the series of compounds obtained from the aconitine mixture. The bioavailability score depends on the Lipinski rule [65], and repeated violations of the parameters that form this rule cause the bioavailability score to decrease. At the same time, the bioavailability of 0.55, as shown by most compounds in the series, means that at physiological pH, 55% of the compound is expected to reach the circulation in unchanged or active form [69]. The results of the bioavailability score can be identified in Figure 4.

Quercitin, genistein, and naringin represent only part of the natural compounds with an excellent role in enhancing the bioavailability of medicines [70].

AC, hypaconitine, mesaconitine, chlorogenic acid, hyperoside, rutin, and naringin are studied compounds that present a low bioavailability score, and the violation of all studied drug design rules can also be observed. This can be determined by the molecular mass and volume of these compounds. 

### 2.2. Identification of Physico-Chemical Properties for the Pharmacological Profile

The pharmacological profile of the compounds can be identified in Table 2. The results indicate that flexibility is high, especially in the series of compounds from the alkaloid class—aconitine (11), hypaconitine (10), mesaconitine (10), gallic acid (6), rutin (6), and naringin (6) all show a high flexibility; on the other hand, for compounds such as catechin, epicatechin, delphinidin, quercetin, naringenin, and genistein, the flexibility is low.

The compounds have an average hydrophobicity, this parameter varying between −1.96 (malvidin) and 4.31 (hypaconitine), and the compounds have an average hydrophilic character.

The TPSA in drug design rules can be associated with bioavailability, the optimal value of this parameter being between 20 and 130 Å 2 [68].

### 2.3. Pharmacokinetic, Pharmacogenomic Profile, and Toxicity of Natural Compounds (ADME-Tox)

The predictive results for the ADME-Tox profile presented in Table 3 show that compounds with good absorption at the intestinal level (exception—magnoflorine). Oral bioavailability is increased for ferric acid, caffeic acid, delphinidin, and syringic acid. Permeability at the level of the blood–brain barrier (BBB) is increased for AC, hypaconitine, mesaconitine, magnoflorine, gallic acid, ferulic acid, malvidin, and syringic acid, and the studied compounds are not inhibitors for OCT2/OCT1 receptors.

The predictive results in the case of the pharmacogenomic profile (Table 4) show that the compounds do not act as CYP3A4 cytochrome inhibitors; instead, they are substrates for this cytochrome (aconitine, hypaconitine, mesaconitine, magnoflorine, chlorogenic acid, hyperoside, rutin, naringin, and quercitrin). For cytochrome CYP2C9, most of the compounds act as inhibitors but not as substrates, and a reduced activity as a substrate/inhibitor is recorded at the site of cytochrome CYP2D6.

The results presented in Table 5 show that most of the compounds do not induce mutagenicity (Ames negative), do not develop toxic character for birds and bees, and are toxic for fish.

At the level of the human body, these compounds develop hepatotoxicity (exceptions: hypaconitine, catechin, ferulic acid, caffeic acid, chlorogenic acid, epicatechin, coumaric acid, and syringic acid), and they are toxic at the mitochondrial level (exceptions: gallic acid, ferulic acid, caffeic acid, coumaric acid, syringic acid, rutin, and naringin).

The compounds do not show nephrotoxicity (exceptions: daidzein coumaric acid delphinidin) and cardiac toxicity. However, most show toxicity at the mitochondrial level (exceptions: gallic acid, caffeic acid, ferulic acid, and coumaric acid).

### 2.4. Pharmacodynamics Profiles of Studied Compounds

The data obtained after running SwissTargetPrediction are presented in Table 6. From a pharmacokinetic point of view, there is a high probability that the compounds thus analyzed bind to different CA isoforms or aldose reductase. 

### 2.5. Molecular Docking Results 

A compound may bind to a specific target if the estimated free energy of binding is lower than −6 Kcal/mol [71,72]. The molecular docking results indicate that quercetin (−7.85 kcal/mol) has the LEFEB when it interacts with AKR1B1 (Table 7). Caffeic acid has an LEFEB of −6.21 kcal/mol, indicating a potential binding interaction with AKR1B1 (Table 7).

According to our molecular docking predictions, chlorogenic acid, coumaric acid, hyperoside, and rutin do not bind to AKR1B1 (Table 7).

## 3. Materials and Methods 

### 3.1. Molecular Modelling of Chemical Compounds

In the first stage, the compounds identified in the bioinformatics databases to select their most suitable formats for the bioinformatics study.

The study analyses twenty-one natural compounds obtained by HPLC from a mixture of aconitine. The separate effect of these chemical structures are analyzed bioinformatically to identify the main medicinal compound. The effect we are looking for in the analyzed compounds is anti-inflammatory, antineoplastic and, more than that, the involvement of the compounds in the nervous system.

Studies in the literature describe the usefulness of polyphenols and some alkaloids in the central nervous system, having a beneficial effect in a series of diseases from anxiety, depression, Alzheimer’s disease, and Parkinson’s disease. Studies suggest the neuroprotective effect of the compounds by reducing inflammation in the brain and by neutralizing oxidases [18,28,29,30,34,73,74].

The SMILES file and the molecular weight of the compounds obtained from the PubChem database can be found in Table 8.

### 3.2. Prediction of Compounds Drug- and Lead-Likeness Features

The rules of drug design as well as the bioavailability score represent a useful tool in predicting the character of a compound to be a medicine. Natural compounds should meet a series of chemical structure design rules to be able to act optimally in the body. The rule of Lipinski [65], Veber [75], Ghose [76], and Egan [67] can be predicted with online tools such as SwissADME [68]. Although the drug design rules are very well calibrated and quite permissive, there are several drugs under study that violate these rules [77]. The Lipinski rule provides for a molecular mass lower than 500 Daltons, a maximum of 10 donor hydrogen bonds, a maximum of 5 acceptor hydrogen bonds, and a log octanol/water (Log P(o/w)) lower than 5 [50,65].

Ghose’s rule shows a molecular mass value between 160 and 480 Daltons, but the Log P(o/w) value must be between 0.4 and 5.6. In addition to Lipinski’s rule, Ghose’s rule also considers the refractivity, which must be between 40 and 130, and the total number of atoms, which must be between 40 and 130, as a molecular descriptor [68,78].

The data used were processed by online bioinformatics software that presents several predictive parameters. These calculation methods use algorithms based on vast training sets and cross-validation accuracy. For example, with regard to SwissADME, the BBB (blood–brain barrier) model was built starting from a training set of 260 permeable or impermeable molecules, obtaining an accuracy of 88% [68].

### 3.3. Identification of Important Physico-Chemical Properties for the Pharmacological Profile

The chemical structures, in SMILES format, were loaded into the MOE software and converted to mol2 files [79]. These files were used for the calculation of physicochemical properties such as flexibility expressed in the number of rotatable bonds, refractivity (Å2), polar molecular surface area (Å2), hydrophobicity, and water solubility (Table 3). 

The chemical structure of the compounds influences their pharmacological activity [80]. The three-dimensional arrangement of the atoms in the molecule can play an important role in the activity of the compounds both in the accessibility to the active sites and for the interaction of the drug with the targeted receptor [18]. The polar molecular surface shows the effective space occupied by the atoms of the molecule [81]. This property is useful in predicting the accessibility of the compound [68].

Flexibility is a characteristic often expressed by the number of their rotatable bonds, being the most common descriptor predicted with the help of bioinformatics tools. From the point of view of flexibility, the studied molecule should not have more than nine rotatable bonds [82].

Solubility (lipophilicity) is the most important physical property of the drug, having a special role in absorption, distribution, and elimination [83].

The partition coefficient (LogP) represents the hydrophobic character and is calculated bioinformatically based on the different solubility of the compound between the polar and the non-polar phase. Hydrophobicity is used to estimate the distribution of drugs in the body [84].

The molecular refraction represents the real volume of the molecules, but also the forces acting on the interaction between the drug and the receptor. This molecular descriptor is calculated by the sum of the atomic refraction of each atom in the molecule [85].

### 3.4. Evaluation of the Pharmacokinetic and Pharmacogenomic Profile of Natural Compounds

pkCSM is a pharmacokinetic properties prediction platform composed of 28 regression and classification models. The models used for prediction with this web server use a Pearson correlation with a coefficient between 0.6 and 0.9 and cross-validation of the data set [86].

An important part of this study consisted of evaluating the pharmacokinetic profile, pharmacogenomics, and toxicity. Absorption, Distribution, Metabolism, Excretion and Toxicity (ADME-T) properties were expressed as intestinal absorption (human), oral bioavailability (human), permeability of the compounds at the level of the blood–brain barrier, percentage of binding to plasma proteins, and inhibition of the OCT receptor at the renal level [87].

Intestinal absorption (human) predicts the proportion of compounds that can be absorbed in the human small intestine. Bioavailability represents the ratio between the amount of active substance administered and the amount absorbed that manifests its biological effect [50]. Permeability at the level of the blood–brain barrier (BBB) represents an important parameter for minimizing the side effects and toxicity of a compound [88]. Computational algorithms predict the ability of a compound to penetrate the BBB [50]. The binding percentage to the plasma proteins is a good indicator of the drugs; they bind to the circulating proteins in the blood and thus cross the cell membranes more easily [18]. The substrate OCT1 (Organic cation transporter (1) is a hepatic transporter with a role in the hepatic clearance of drugs, and OCT2 (Organic cation transporter (2) is a renal transporter with a role in the elimination of drugs and endogenous compounds. Both OCT1 and OCT2 have a special role in the body’s clearance [18].

Cytochrome P450 is a detoxification enzyme in the body, which is found in the liver and helps to excrete xenobiotics from the body, but it can also deactivate many drugs. Inhibitors of these enzymes affect the metabolism of drugs and therefore the ability of compounds to inhibit cytochrome P450 is evaluated. Cytochrome P450 substrate or activator predicts the likelihood that a molecule will be metabolized by P450. CYP1A2, CYP2C19, CYP2C9, CYP2D6, and CYP3A4 are cytochrome P450 isoforms and can be either inhibitors or substrates for different chemical compounds [18,86].

### 3.5. Toxicity Profile of Natural Compounds

A significant number of items representing toxicity were analyzed for natural compounds—Ames (mutagenesis), carcinogenicity, toxicity to different species (crustacea, bees, fish), nephrotoxicity, hepatotoxicity, cardiotoxicity, mitochondrial toxicity, toxicity to nuclear receptors, and so on [80]. 

Ames toxicity for a compound follows its mutagenic character; if it turns out to be positive, then in this sense, the compound is mutagenic [18,89].

Carcinogenicity is predicted to identify the possible carcinogenic character of a compound [80].

Aquatic toxicity for fish and crustaceans is also a common indicator for toxicity, with positive compounds for this category inhibiting the growth of half of the studied fish/crustacean population. This type of toxicity refers to the toxicity of the environment, with some drugs not being completely metabolized, being eliminated in their active state or their active metabolites and ending up in domestic water through excretion and in the soil, thus affecting the balance of the environment [80,86].

### 3.6. Pharmacodynamics Profile of Compounds

The pharmacological effects of AC and its derivatives are presented in Table 1. AC binds to certain receptors in the body to determine the beneficial effect for the body [90,91]. To model compounds, it is necessary to know the molecular target to which these compounds can bind. That is why we will use molecular target prediction software for the studied compounds.

SwissTargetPrediction is an online bioinformatics tool hosted by the Swiss Institute of Bioinformatics SIB that predicts their protein targets based on the similarity of small molecules, based on a library of 370,000 active substances on known targets [92].

### 3.7. Molecular Modeling

The structure of quercetin, caffeic acid, chlorogenic acid, coumaric acid, hyperoside, and rutin was imported from PubChem and optimized using our usual protocol [93]. The molecules were minimized using Hamiltonian Forcefield MMFF94x at a 0.05 gradient with Gasteiger (PEOE) partial charges [94]. The structures were exported as “.mol2” and converted as pdbqt for the molecular docking analysis using Open Babel software [95].

The structure of AKR1B1 was imported from the RCSB Protein Data Bank (PDB ID: 2ACQ) [96] and optimized for the molecular docking studies according to our usual protocol, we deleted the water molecules and the residues, added the hydrogen atoms, merged the non-polar hydrogen atoms, and applied the Kollman charges [47,72].

### 3.8. Molecular Docking

Using AutoDock 4.2.6 and following our standard procedure (we used the Lamarckian Genetic Algorithm search parameter, and we generated 100 runs for each protein-ligand complex), we predicted the interaction between natural compounds and selected target proteins [71,72,97]. We ran blind docking simulations with the following grid-box parameters:

The number of specified grid points is 126 (x, y, z); the grid point spacing is 0.375 angstroms; and the coordinates of the central grid point of maps are 15.064, 32.982, and 68.073).

## 4. Discussion

Active compounds from *Aconitum toxicum Rchb.*, *Anemone nemorosa L.*, *and Helleborus odorus* Waldst. & Kit. ex Willd were analyzed from a bioinformatics point of view, both pharmacokinetically and pharmacodynamically.

Alkaloids with more complex chemical structures (AC, hypaconitine, mesaconitine, hyperoside, rutin, and naringin) show violations of drug design rules (Lipinski, Ghose, Egan, and Muegge rules), while the compounds magnoflorine, catechin, epicatechin, daidzein, malvidin, quercetin, naringenin, and genistein do not violate any of the drug design rules. The bioavailability score varies between 0.11 (chlorogenic acid) and 0.85 (ferulic acid and coumaric acid).

The pharmacological profile of a compound shows high values of flexibility and refractivity, and TPSA can be observed for alkaloids AC, hypaconitine, and mesaconitine, but also for hyperoside, naringin, and rutin.

All the analyzed compounds can be absorbed at the intestinal level, while only ferulic acid, delphinid, caffeic acid, daidzein, and syringic acid are bioavailable.

AC, hypaconitine, mesaconitine, magnoflorine, gallic acid and ferulic acid, malvidin, and syringic acid can cross the BBB. All the compounds mentioned above have a higher binding affinity at the level of plasma proteins, the rest of the compounds being predicted to circulate freely in the blood in higher proportions. Additionally, except for magnoflorine (OCT1 inhibitor), no compound is an inhibitor of OCT1 and OCT2.

From the point of view of mutagenesis and toxicity to aquatic crustaceans and birds, predominantly, the compounds are predicted to be non-toxic. Instead, the predictions show that all the compounds analyzed are toxic to fish, and most of them show hepatotoxicity and toxicity at the level of mitochondria. These data correlate with the data from the literature, from the study by Wang et al., who stated that aconitine affects signaling pathways and mitochondria, causing apoptosis. Mitochondrial damage can have multiple negative effects based on the length of time exposure to the toxic compound. By treating H9c2 hippocampal cells with aconitine, mitochondrial dysfunction was induced, cytochrome Bax was upregulated, Caspaseno3 was cleaved, and Bcl 2 was decreased, thus providing a possible mechanism of apoptosis mediated by mitochondria [98]. In the case of the study by Ravindran et al., apoptosis depended both on the compound concentration to which the cells were exposed and on their incubation time with the actual compound [99].

For hepatotoxicity, there is not much evidence in humans, but studies on animal models such as rats show a high tendency to accumulate alkaloids in organs such as the liver and kidneys, but this is achieved after long-term oral administration. The study by Xiaoyu Ji et al. does not specify the identified period that can determine the accumulation of these alkaloids in animal tissues. At the same time, the optimal administration time and the recommended dose for rats are not known, so as not to determine liver damage induced by the consumption of the medicinal compound [12].

Making the prediction of molecular targets, we observe a prevalence of carbonic anhydrase to which the compounds bind with high affinity [100]. In these predictions, several isoforms of the enzyme with the role of regulating the acid–base balance appear, but CA II and CA VII are the most frequently encountered.

Supuran’s review showed a high affinity of coumarin for CA, inhibiting their activity. These natural compounds have a binding affinity to CA in areas inaccessible for other compounds [52].

Naringin is a natural phenol, and although it was predicted to have a binding affinity to cytochrome P450, studies in the literature prove the important inhibitory activity of naringin not only on the CA II isoform, but also on other important receptors in the central nervous system such as αnoglucosidase (αnoGly), acetylcholinesterase (AChE), and butyrylcholinesterase (BChE) [101]. Caffeic acid and other natural phenols have been identified as CA II inhibitors [102,103].

**Figure 5:**
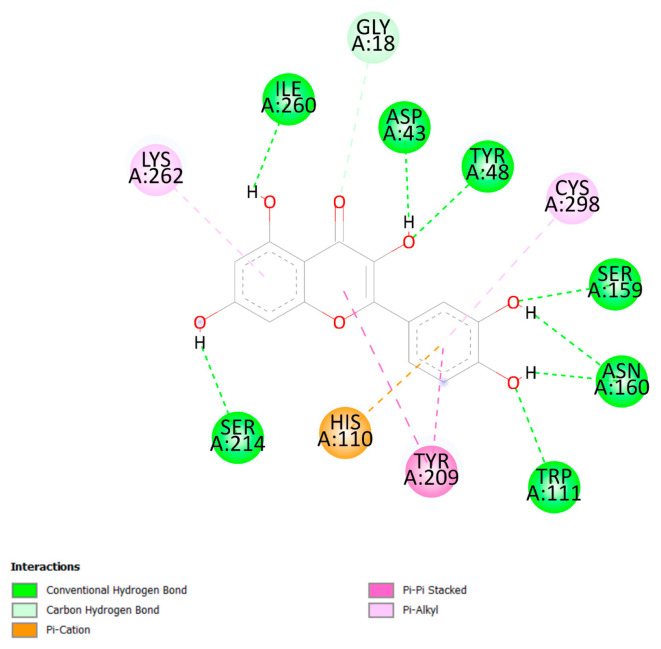
A 2D visualization of the interactions between quercetin and AKR1B1 amino acid residues Discovery Studio Visualizer was used to obtain the image [104].

Quercetin has the lowest predicted energy of binding when compared to the other evaluated natural compounds. According to our computer simulations, quercetin and AKR1B1 interact favorably and form the conventional hydrogen-bound, carbon-hydrogen-bound, pi-cation, pi-pi stacked, and pi-alkyl interactions (Figure 5).

## 5. Conclusions

The studies in the literature confirm the predictions made with the bioinformatics tools for the compounds obtained from extracts of three Ranunculaceae species, *Aconitum toxicum* Rchb., *Anemone nemorosa* L., and *Helleborus odorus* Waldst. & Kit. ex Willd. by the HPLC method. The analyzed compounds that inhibit CA II can be used for testing new treatment modalities in cases of glaucoma, neoplasms, and neurodegenerative diseases, diseases in which a great improvement was observed following the inhibition of the CA enzyme [105].

The results obtained following the prediction of molecular targets are not significant for AC, gallic acid, delphinidin, malvidin, and mesaconitine.

Magnoflorine and rutin have targets on metabotropic receptors, and genistein, which is a non-specific compound, has a probability of binding both on nuclear and metabotropic receptors.

The results obtained from this study show the prevalence of natural compounds obtained from *Aconitum toxicum* Rchb., *Anemone nemorosa* L., and *Helleborus odorus* Waldst. & Kit. ex Willd of binding at the level of the carbonic anhydrase family and the aldo-keto reductase family. Quercitin presented the best binding energy and the best values of the molecular descriptors. At the same time, additional studies are needed on AC, hypaconitine, magnoflorine, gallic acid, ferulic acid, malvidin and syringic acid due to the permeability of these compounds to cross the blood–brain barrier.

## Figures and Tables

**Table 2:** The pharmacological profile of natural compounds using descriptors such as flexibility, refractivity, topological polar surface area (TPSA), hydrophobicity, and solubility.

Compound	Flexibility	Refractivity	TPSA	Hydrophobicity	Solubility
Aconitine	11	165.5	153.45	3.61	−3.39
Hypaconitine	10	159.53	133.22	4.3	−3.65
Mesaconitine	10	160.69	153.45	3.56	−3.14
Magnoflorine	2	101.87	58.92	−0.66	−3.91
Gallic acid	6	119.15	64.99	3.97	−6.25
Catechin	1	74.33	110.38	1.47	−2.22
Ferulic acid	3	51.63	66.76	1.62	−2.11
Caffeic acid	2	47.16	77.76	0.97	−1.89
Chlorogenic acid	5	83.5	164.75	0.96	−1.62
Epicatechin	1	74.33	110.38	1.47	−2.22
Delphinidin	1	75.26	134.19	1.35	−2.35
Coumaric acid	2	45.13	57.53	0.95	−2.02
Daidzein	1	71.97	70.67	1.77	−3.53
Hyperoside	4	110.16	210.51	2.11	−3.04
Rutin	6	141.38	269.43	1.58	−3.3
Naringin	6	134.91	225.06	2.38	−2.98
Malvidin	3	87.13	112.52	−1.96	−3.6
Quercetin	1	78.03	131.36	1.63	−3.16
Naringenin	1	71.57	86.99	1.75	−3.49
Genistein	1	73.99	90.9	1.91	−3.72
Syringic acid	3	48.41	75.99	1.54	−1.84

**Table 3:** Pharmacokinetic properties of natural compounds.

Compound	Intestinal Absorption (Human)	Bioavailability	BBB Permeability	Fraction Unbound (Human)	OCT2 Inhibitor	OCT1 Inhibitor
AC	yes	no	yes	0.52	no	no
Hypaconitine	yes	no	yes	0.78	no	no
Mesaconitine	yes	no	yes	0.53	no	no
Magnoflorine	no	no	yes	0.19	no	yes
Gallic acid	yes	no	yes	0.99	no	no
Catechin	yes	no	no	1.01	no	no
Ferulic acid	yes	yes	yes	0.72	no	no
Caffeic acid	yes	yes	no	0.73	no	no
Chlorogenic acid	yes	no	no	0.63	no	no
Epicatechin	yes	no	no	1.01	no	no
Delphinidin	yes	yes	no	0.85	no	no
Coumaric acid	yes	no	no	0.50	no	no
Daidzein	yes	yes	no	0.96	No	no
Hyperoside	yes	no	no	0.79	no	no
Rutin	yes	no	no	0.96	no	no
Naringin	yes	no	no	0.73	no	no
Malvidin	yes	no	yes	0.88	no	no
Quercetin	yes	no	no	1.16	no	no
Naringenin	yes	no	no	0.93	no	no
Genistein	yes	no	no	1.09	no	no
Syringic acid	yes	yes	yes	0.55	no	no

**Table 4:** Pharmacogenomic profile predicted for analyzed compounds.

Compounds	CYP1A2 Inhibitor	CYP2C19 Inhibitor	CYP2C9 Inhibitor	CYP2C9 Substrate	CYP2D6 Inhibitor	CYP2D6 Substrate	CYP3A4 Inhibitor	CYP3A4 Substrate
AC	no	no	no	no	no	no	no	yes
Hypaconitine	no	no	no	no	no	no	no	yes
Mesaconitine	no	no	no	no	no	no	no	yes
Magnoflorine	no	no	no	no	no	yes	no	yes
Gallic acid	no	yes	yes	no	no	no	no	no
Catechin	no	no	no	no	no	yes	no	no
ferulic acid	no	no	no	no	no	no	no	no
Caffeic acid	no	no	no	no	no	no	no	no
Chlorogenic acid	no	no	no	yes	no	no	no	yes
Epicatechin	no	no	no	no	no	yes	no	no
Delphinidin	yes	yes	yes	no	no	no	yes	no
Coumaric acid	no	no	no	no	no	no	no	no
Daidzein	yes	yes	yes	no	no	no	no	no
Hyperoside	no	no	no	no	no	no	no	yes
Rutin	no	no	no	no	no	no	no	yes
Naringin	no	no	no	no	no	no	no	yes
Malvidin	yes	yes	yes	no	no	no	yes	no
Quercetin	yes	no	no	no	no	no	yes	yes
Naringenin	yes	yes	yes	no	no	no	yes	no
Genistein	yes	yes	yes	no	no	no	yes	no
Syringic acid	no	no	no	no	no	no	no	no

**Table 5:** Natural compounds predicted toxicity.

Compounds	Ames Mutagenesis	Avian Toxicity	Crustacea Aquatic Toxicity	Toxicities Fish	Hepatotoxicity	Mitochondrial Toxicity
AC	no	no	yes	yes	yes	yes
Hypaconitine	no	no	yes	yes	no	yes
Mesaconitine	no	no	no	yes	yes	yes
Magnoflorine	no	no	yes	yes	yes	yes
Gallic acid	no	no	no	yes	yes	no
Catechin	yes	no	yes	yes	no	yes
Ferulic acid	no	no	no	yes	no	no
Caffeic acid	no	no	no	yes	no	no
Chlorogenic acid	no	no	no	yes	no	yes
Epicatechin	yes	no	yes	yes	no	yes
Delphinidin	no	no	no	yes	yes	yes
Coumaric acid	no	no	no	yes	no	no
Daidzein	no	no	no	yes	yes	no
Hyperoside	yes	no	no	yes	yes	yes
Rutin	yes	no	no	yes	yes	no
Naringin	no	no	no	yes	yes	no
Malvidin	no	no	yes	yes	yes	yes
Quercetin	yes	no	no	yes	yes	yes
Naringenin	no	no	no	yes	yes	yes
Genistein	no	no	no	yes	yes	yes
Syringic acid	no	no	no	yes	no	no

**Table 6:** Targets obtain using SwissTargetPrediction tool.

Target Name	Target Abbreviation	Binding Probability
Caffeic acid		
Carbonic anhydrase II	CA2	0.72
Arachidonate 5nolipoxygenase	ALOX5	0.72
Carbonic anhydrase VII	CA7	0.72
Carbonic anhydrase I	CA1	0.7
Carbonic anhydrase VI	CA6	0.7
Chlorogenic acid		
Aldose reductase	AKR1B1	0.87
Aldonoketo reductase family 1 member B10	AKR1B10	0.74
Coumaric acid		
Aldose reductase	AKR1B1	1
Carbonic anhydrase II	CA2	1
Carbonic anhydrase VII	CA7	1
Estrogen receptor beta	ESR2	1
Carbonic anhydrase I	CA1	1
Carbonic anhydrase III	CA3	1
Carbonic anhydrase VI	CA6	1
Carbonic anhydrase XII	CA12	1
Carbonic anhydrase XIV	CA14	1
Carbonic anhydrase IX	CA9	1
Carbonic anhydrase IV	CA4	1
Carbonic anhydrase VB	CA5B	1
Carbonic anhydrase VA	CA5A	1
Ferulic acid		
Carbonic anhydrase II	CA2	0.93
Carbonic anhydrase VII	CA7	0.93
Carbonic anhydrase I	CA1	0.93
Carbonic anhydrase VI	CA6	0.93
Carbonic anhydrase XII	CA12	0.9
Carbonic anhydrase XIV	CA14	0.901
Carbonic anhydrase IX	CA9	0.901
Carbonic anhydrase VA	CA5A	0.9
Gallic acid without statistical significance		
Syringic acid		
Carbonic anhydrase II	CA2	1
Carbonic anhydrase VII	CA7	1
Carbonic anhydrase I	CA1	1
Carbonic anhydrase III	CA3	1
Carbonic anhydrase VI	CA6	1
Carbonic anhydrase XII	CA12	1
Carbonic anhydrase XIV	CA14	1
Carbonic anhydrase IX	CA9	1
Carbonic anhydrase VA	CA5A	1
AC without statistical significance		
Daidzein		
Aldehyde dehydrogenase	ALDH2	1
Estrogen receptor alpha	ESR1	1
Carbonic anhydrase VII	CA7	1
Estrogen receptor beta	ESR2	1
Carbonic anhydrase XII	CA12	1
Carbonic anhydrase IV	CA4	1
Delfinidin without statistical significance		
Genistein		
ThromboxanenoA synthase	TBXAS1	1
Monoamine oxidase A	MAOA	1
Epidermal growth factor receptor erbB1	EGFR	1
Estrogen receptor alpha	ESR1	1
Maltasenoglucoamylase	MGAM	1
Serotonin 2a (5noHT2a) receptor	HTR2A	1
Serotonin 2c (5noHT2c) receptor	HTR2C	1
Adenosine A1 receptor (by homology)	ADORA1	1
Estrogen receptor beta	ESR2	1
Adenosine A2a receptor	ADORA2A	1
Estradiol 17nobetanodehydrogenase 1	HSD17B1	1
Estrogennorelated receptor alpha	ESRRA	1
Estrogennorelated receptor beta	ESRRB	1
ATPnobinding cassette subnofamily G member 2	ABCG2	1
Hypaconitine		
Dopamine transporter (by homology)	SLC6A3	0.093576
HERG	KCNH2	0.074565
Hyperoside		
Aldose reductase	AKR1B1	1
Carbonic anhydrase II	CA2	1
Carbonic anhydrase VII	CA7	1
Carbonic anhydrase XII	CA12	1
Carbonic anhydrase IV	CA4	1
Magnoflorine		
Dopamine D2 receptor	DRD2	0.548778
Neuronal acetylcholine receptor; alpha4/beta2	CHRNA4 CHRNB2	0.390156
Dopamine D3 receptor	DRD3	0.306473
Malvidin without statistical significance		
Glyoxalase I	GLO1	0.128899
Xanthine dehydrogenase	XDH	0.112748
Mesaconitine without statistical significance		
Naringenin		
Cytochrome P450 19A1	CYP19A1	0.91
Carbonic anhydrase VII	CA7	0.91
Multidrug resistance no associated protein 1	ABCC1	0.91
Estradiol 17nobetanodehydrogenase 1	HSD17B1	0.91
Carbonic anhydrase XII	CA12	0.91
Test is no specific androgen no binding protein	SHBG	0.91
Carbonic anhydrase IV	CA4	0.91
Cytochrome P450 1B1	CYP1B1	0.9
Carbonyl reductase [NADPH] 1	CBR1	0.9
Naringin		
Cytochrome P450 19A1	CYP19A1	1
Quercetin		
NADPH oxidase 4	NOX4	1
Vasopressin V2 receptor	AVPR2	1
Aldose reductase	AKR1B1	1
Xanthine dehydrogenase	XDH	1
Monoamine oxidase A	MAOA	1
Insulinnolike growth factor I receptor	IGF1R	1
Rutin		
NeuromedinnoU receptor 2	NMUR2	1
Alphano2a adrenergic receptor	ADRA2A	1
Adrenergic receptor alphano2	ADRA2C	1
Acetylcholinesterase	ACHE	1
Aldose reductase	AKR1B1	0.60286

**Table 7:** The LEFEB between AKR1B1 and quercetin, caffeic acid, chlorogenic acid, coumaric acid, hyperoside, and rutin.

Compound	LEFEB
Quercetin	−7.85 kcal/mol
Caffeic acid	−6.21 kcal/mol
Chlorogenic acid	−5.81 kcal/mol
Coumaric acid	−5.60 kcal/mol
Hyperoside	−5.59 kcal/mol
Rutin	−4.54 kcal/mol

**Table 8:** Chemical compounds name, SMILES format, Molecular Formula and Molecular Weight of the analyzed structure.

Name	SMILES	Formula	MW (Da)
AC	COCC12CN(CC)C3C4(C2C(OC)C3C2(C3C4CC(C3OC(=O)c3ccccc3)(C(C2O)OC)O)OC(=O)C)C(CC1O)OC	C34H47NO11	645.74
Hypaconitine	COCC12CCC(C34C2C(OC)C(C3N(C1)C)C1(C2C4CC(C2OC(=O)c2ccccc2)(C(C1O)OC)O)OC(=O)C)OC	C33H45NO10	615.71
Mesaconitine	COCC12CN(C)C3C4(C2C(OC)C3C2(C3C4CC(C3OC(=O)c3ccccc3)(C(C2O)OC)O)OC(=O)C)C(CC1O)OC	C33H45NO11	631.71
Magnoflorine	COc1ccc2c(c1O)c1c(O)c(OC)cc3c1C(C2)[N+](C)(C)CC3	C20H24NO4+	342.41
Gallic acid	O=C(c1cc(O)c2c(c1)OC(O2)(c1ccccc1)c1ccccc1)OCc1ccccc1	C27H20O5	424.44
Catechin	Oc1cc2OC(c3ccc(c(c3)O)O)C(Cc2c(c1)O)O	C15H14O6	290.27
Ferulic acid	COc1cc(C=CC(=O)O)ccc1O	C10H10O4	194.18
Caffeic acid	OC(=O)C=Cc1ccc(c(c1)O)O	C9H8O4	180.16
Chlorogenic acid	O=C(OC1CC(O)(CC(C1O)O)C(=O)O)C=Cc1ccc(c(c1)O)O	C16H18O9	354.31
Epicatechin	Oc1cc2OC(c3ccc(c(c3)O)O)C(Cc2c(c1)O)O	C15H14O6	290.27
Delfinidin	O=c1cc2oc(c3cc(O)c(c(c3)O)[O-])c(cc2c(c1)O)O	C15H9O7-	301.23
Coumaric acid	OC(=O)C=Cc1ccc(cc1)O	C9H8O3	164.16
Daidzein	Oc1ccc(cc1)c1coc2c(c1=O)ccc(c2)O	C15H10O4	254.24
Hyperoside	OCC1OC(Oc2c(oc3c(c2=O)c(O)cc(c3)O)c2ccc(c(c2)O)O)C(C(C1O)O)O	C21H20O12	464.38
Rutin	Oc1cc(O)c2c(c1)oc(c(c2=O)OC1OC(COC2OC(C)C(C(C2O)O)O)C(C(C1O)O)O)c1ccc(c(c1)O)O	C27H30O16	610.52
Naringin	OCC1OC(Oc2cc(O)c3c(c2)OC(CC3=O)c2ccc(cc2)O)C(C(C1O)O) OC1OC(C)C(C(C1O)O)O	C27H32O14	580.53
Malvidin	COc1cc(cc(c1O)OC)c1[o+]c2cc(O)cc(c2cc1O)O	C17H15O7+	331.3
Quercetin	Oc1cc(O)c2c(c1)oc(c(c2=O)O)c1ccc(c(c1)O)O	C15H10O7	302.24
Naringenin	Oc1ccc(cc1)C1CC(=O)c2c(O1)cc(cc2O)O	C15H12O5	272.25
Genistein	Oc1ccc(cc1)c1coc2c(c1=O)c(O)cc(c2)O	C15H10O5	270.24
Syringic acid	COc1cc(cc(c1O)OC)C(=O)O	C9H10O5	198.17

## Data Availability

Not applicable.

## References

[1] Li S., Yu L., Shi Q., Liu Y., Zhang Y., Wang S., Lai X. (2022). An Insight into Current Advances on Pharmacology, Pharmacokinetics, Toxicity and Detoxification of Aconitine. Biomed. Pharmacother..

[2] Xie Y., Mai C.-T., Zheng D.-C., He Y.-F., Feng S.-L., Li Y.-Z., Liu C.-X., Zhou H., Liu L. (2021). Wutou Decoction Ameliorates Experimental Rheumatoid Arthritis via Regulating NF-KB and Nrf2: Integrating Efficacy-Oriented Compatibility of Traditional Chinese Medicine. Phytomedicine.

[3] Wang J.-J., Lou H.-Y., Liu Y., Han H.-P., Ma F.-W., Pan W.-D., Chen Z. (2022). Profiling Alkaloids in Aconitum Pendulum N. Busch Collected from Different Elevations of Qinghai Province Using Widely Targeted Metabolomics. Phytochemistry.

[4] Mares C., Udrea A.-M., Buiu C., Staicu A., Avram S. (2023). Therapeutic Potentials of Aconite-like Alkaloids - Bioinformatics and Experimental Approaches. Mini Rev. Med. Chem..

[5] Liao Y.-P., Shen L.-H., Cai L.-H., Chen J., Shao H.-Q. (2022). Acute Myocardial Necrosis Caused by Aconitine Poisoning: A Case Report. World J. Clin. Cases.

[6] Chan T.Y.K. (2009). Aconite Poisoning. Clin. Toxicol. Phila. Pa.

[7] Mi L., Li Y.-C., Sun M.-R., Zhang P.-L., Li Y., Yang H. (2021). A Systematic Review of Pharmacological Activities, Toxicological Mechanisms and Pharmacokinetic Studies on Aconitum Alkaloids. Chin. J. Nat. Med..

[8] Wang X., Lin Y., Zheng Y. (2020). Antitumor Effects of Aconitine in A2780 Cells via Estrogen Receptor Β-mediated Apoptosis, DNA Damage and Migration. Mol. Med. Rep..

[9] Singhuber J., Zhu M., Prinz S., Kopp B. (2009). Aconitum in Traditional Chinese Medicine—A Valuable Drug or an Unpredictable Risk?. J. Ethnopharmacol..

[10] Gao X., Hu J., Zhang X., Zuo Y., Wang Y., Zhu S. (2018). Research Progress of Aconitine Toxicity and Forensic Analysis of Aconitine Poisoning. Forensic Sci. Res..

[11] Hu J., Wu Q., Li Q., Lv T., Peng T.-F., Yin S., Jin H.-Z. (2023). Antinociceptive Diterpenoid Alkaloids from the Roots of Aconitum Austroyunnanense. J. Asian Nat. Prod. Res..

[12] Ji X., Yang M., Or K.H., Yim W.S., Zuo Z. (2019). Tissue Accumulations of Toxic Aconitum Alkaloids after Short-Term and Long-Term Oral Administrations of Clinically Used Radix Aconiti Lateralis Preparations in Rats. Toxins.

[13] Lu H., Mei L., Guo Z., Wu K., Zhang Y., Tang S., Zhu Y., Zhao B. (2022). Hematological and Histopathological Effects of Subacute Aconitine Poisoning in Mouse. Front. Vet. Sci..

[14] Deng J., Han J., Chen J., Zhang Y., Huang Q., Wang Y., Qi X., Liu Z., Leung E.L.-H., Wang D. (2021). Comparison of Analgesic Activities of Aconitine in Different Mice Pain Models. PLoS ONE.

[15] Zhang Y., Zong X., Wu J.-L., Liu Y., Liu Z., Zhou H., Liu L., Li N. (2020). Pharmacokinetics and Tissue Distribution of Eighteen Major Alkaloids of Aconitum Carmichaelii in Rats by UHPLC-QQQ-MS. J. Pharm. Biomed. Anal..

[16] PubChem Aconitine. https://pubchem.ncbi.nlm.nih.gov/compound/245005.

[17] Seiman D.D., Batalu A., Seiman C.D., Ciopec M., Udrea A.M., Motoc M., Negrea A., Avram S. (2018). Pharmacological Effects of Natural Compounds Extracted from Urtica Dioica Evaluated by in Silico and Experimental Methods. Rev Chim Buchar..

[18] Avram S., Stan M.S., Udrea A.M., Buiu C., Boboc A.A., Mernea M. (2021). 3D-ALMOND-QSAR Models to Predict the Antidepressant Effect of Some Natural Compounds. Pharmaceutics.

[19] Iban-Arias R., Sebastian-Valverde M., Wu H., Lyu W., Wu Q., Simon J., Pasinetti G.M. (2022). Role of Polyphenol-Derived Phenolic Acid in Mitigation of Inflammasome-Mediated Anxiety and Depression. Biomedicines.

[20] Zhao W., Wang J., Bi W., Ferruzzi M., Yemul S., Freire D., Mazzola P., Ho L., Dubner L., Pasinetti G.M. (2015). Novel Application of Brain-Targeting Polyphenol Compounds in Sleep Deprivation-Induced Cognitive Dysfunction. Neurochem. Int..

[21] de la Rosa L.A., Alvarez-Parrilla E., Shahidi F. (2011). Phenolic Compounds and Antioxidant Activity of Kernels and Shells of Mexican Pecan (Carya Illinoinensis). J. Agric. Food Chem..

[22] Addepalli V., Suryavanshi S.V. (2018). Catechin Attenuates Diabetic Autonomic Neuropathy in Streptozotocin Induced Diabetic Rats. Biomed. Pharmacother..

[23] Agunloye O.M., Oboh G., Ademiluyi A.O., Ademosun A.O., Akindahunsi A.A., Oyagbemi A.A., Omobowale T.O., Ajibade T.O., Adedapo A.A. (2019). Cardio-Protective and Antioxidant Properties of Caffeic Acid and Chlorogenic Acid: Mechanistic Role of Angiotensin Converting Enzyme, Cholinesterase and Arginase Activities in Cyclosporine Induced Hypertensive Rats. Biomed. Pharmacother..

[24] de Araújo F.F., de Paulo Farias D., Neri-Numa I.A., Pastore G.M. (2021). Polyphenols and Their Applications: An Approach in Food Chemistry and Innovation Potential. Food Chem..

[25] Chandrasekhar Y., Phani Kumar G., Ramya E.M., Anilakumar K.R. (2018). Gallic Acid Protects 6-OHDA Induced Neurotoxicity by Attenuating Oxidative Stress in Human Dopaminergic Cell Line. Neurochem. Res..

[26] Sakalauskas A., Ziaunys M., Smirnovas V. (2020). Gallic Acid Oxidation Products Alter the Formation Pathway of Insulin Amyloid Fibrils. Sci. Rep..

[27] Seo E.-J., Fischer N., Efferth T. (2018). Phytochemicals as Inhibitors of NF-ΚB for Treatment of Alzheimer’s Disease. Pharmacol. Res..

[28] Ogut E., Armagan K., Gül Z. (2022). The Role of Syringic Acid as a Neuroprotective Agent for Neurodegenerative Disorders and Future Expectations. Metab. Brain Dis..

[29] Nouri Z., Fakhri S., El-Senduny F.F., Sanadgol N., Abd-ElGhani G.E., Farzaei M.H., Chen J.-T. (2019). On the Neuroprotective Effects of Naringenin: Pharmacological Targets, Signaling Pathways, Molecular Mechanisms, and Clinical Perspective. Biomolecules.

[30] Enogieru A.B., Haylett W., Hiss D.C., Bardien S., Ekpo O.E. (2018). Rutin as a Potent Antioxidant: Implications for Neurodegenerative Disorders. Oxid. Med. Cell. Longev..

[31] Xie J., Guo L., Pang G., Wu X., Zhang M. (2011). Modulation Effect of Semen Ziziphi Spinosae Extracts on IL-1β, IL-4, IL-6, IL-10, TNF-α and IFN-γ in Mouse Serum. Nat. Prod. Res..

[32] Li B., Han L., Cao B., Yang X., Zhu X., Yang B., Zhao H., Qiao W. (2019). Use of Magnoflorine-Phospholipid Complex to Permeate Blood-Brain Barrier and Treat Depression in the CUMS Animal Model. Drug Deliv..

[33] Yi J.H., Moon S., Cho E., Kwon H., Lee S., Jeon J., Park A.Y., Lee Y.H., Kwon K.J., Ryu J.H. (2022). Hyperoside Improves Learning and Memory Deficits by Amyloid Β1-42 in Mice through Regulating Synaptic Calcium-Permeable AMPA Receptors. Eur. J. Pharmacol..

[34] Chen L., Zhou Y.-P., Liu H.-Y., Gu J.-H., Zhou X.-F., Yue-Qin Z. (2021). Long-Term Oral Administration of Hyperoside Ameliorates AD-Related Neuropathology and Improves Cognitive Impairment in APP/PS1 Transgenic Mice. Neurochem. Int..

[35] Kim S., Chen J., Cheng T., Gindulyte A., He J., He S., Li Q., Shoemaker B.A., Thiessen P.A., Yu B. (2021). PubChem in 2021: New Data Content and Improved Web Interfaces. Nucleic Acids Res..

[36] Buiu C., Putz M.V., Avram S. (2016). Learning the Relationship between the Primary Structure of HIV Envelope Glycoproteins and Neutralization Activity of Particular Antibodies by Using Artificial Neural Networks. Int. J. Mol. Sci..

[37] Demir Y., Durmaz L., Taslimi P., Gulçin İ. (2019). Antidiabetic Properties of Dietary Phenolic Compounds: Inhibition Effects on α-Amylase, Aldose Reductase, and α-Glycosidase. Biotechnol. Appl. Biochem..

[38] Garbiec E., Cielecka-Piontek J., Kowalówka M., Hołubiec M., Zalewski P. (2022). Genistein-Opportunities Related to an Interesting Molecule of Natural Origin. Mol. Basel Switz..

[39] Zeng X., He L., Wang S., Wang K., Zhang Y., Tao L., Li X., Liu S. (2016). Aconine Inhibits RANKL-Induced Osteoclast Differentiation in RAW264.7 Cells by Suppressing NF-ΚB and NFATc1 Activation and DC-STAMP Expression. Acta Pharmacol. Sin..

[40] Zhang L., Siyiti M., Zhang J., Yao M., Zhao F. (2021). Anti-Inflammatory and Anti-Rheumatic Activities in Vitro of Alkaloids Separated from Aconitum Soongoricum Stapf. Exp. Ther. Med..

[41] Zhu L., Wu J., Zhao M., Song W., Qi X., Wang Y., Lu L., Liu Z. (2017). Mdr1a Plays a Crucial Role in Regulating the Analgesic Effect and Toxicity of Aconitine by Altering Its Pharmacokinetic Characteristics. Toxicol. Appl. Pharmacol..

[42] Çankal D., Akkol E.K., Kılınç Y., İlhan M., Capasso R. (2021). An Effective Phytoconstituent Aconitine: A Realistic Approach for the Treatment of Trigeminal Neuralgia. Mediators Inflamm..

[43] Ji B.-L., Xia L.-P., Zhou F.-X., Mao G.-Z., Xu L.-X. (2016). Aconitine Induces Cell Apoptosis in Human Pancreatic Cancer via NF-ΚB Signaling Pathway. Eur. Rev. Med. Pharmacol. Sci..

[44] Li X.-M., Liu J., Pan F.-F., Shi D.-D., Wen Z.-G., Yang P.-L. (2018). Quercetin and Aconitine Synergistically Induces the Human Cervical Carcinoma HeLa Cell Apoptosis via Endoplasmic Reticulum (ER) Stress Pathway. PLOS ONE.

[45] Guo B. (2011). Effects of Osthole, Psoralen, Aconitine on Breast Cancer MDA-MB-231BO Cell Line Inhibition in Vitro. J. Chin. Integr. Med..

[46] Udrea A.-M., Dinache A., Staicu A., Avram S. (2022). Target Prediction of 5,10,15,20-Tetrakis(4′-Sulfonatophenyl)-Porphyrin Using Molecular Docking. Pharmaceutics.

[47] Tozar T., Santos Costa S., Udrea A.-M., Nastasa V., Couto I., Viveiros M., Pascu M.L., Romanitan M.O. (2020). Anti-Staphylococcal Activity and Mode of Action of Thioridazine Photoproducts. Sci. Rep..

[48] Liu M., Cao Y., Lv D., Zhang W., Zhu Z., Zhang H., Chai Y. (2017). Effect of Processing on the Alkaloids in Aconitum Tubers by HPLC-TOF/MS. J. Pharm. Anal..

[49] Avram S., Milac A.-L., Mihailescu D. (2012). 3D-QSAR Study Indicates an Enhancing Effect of Membrane Ions on Psychiatric Drugs Targeting Serotonin Receptor 5-HT1A. Mol. Biosyst..

[50] Avram S., Buiu C., Duda-Seiman D., Duda-Seiman C., Borcan F., Mihailescu D. (2012). Evaluation of the Pharmacological Descriptors Related to the Induction of Antidepressant Activity and Its Prediction by QSAR/QRAR Methods. Mini Rev. Med. Chem..

[51] Avram S., Mernea M., Limban C., Borcan F., Chifiriuc C. (2020). Potential Therapeutic Approaches to Alzheimer’s Disease By Bioinformatics, Cheminformatics And Predicted Adme-Tox Tools. Curr. Neuropharmacol..

[52] Supuran C.T. (2018). Carbonic Anhydrase Activators. Future Med. Chem..

[53] Supuran C.T. (2010). Carbonic Anhydrase Inhibitors. Bioorg. Med. Chem. Lett..

[54] Avram S., Milac A.L., Carta F., Supuran C.T. (2013). More Effective Dithiocarbamate Derivatives Inhibiting Carbonic Anhydrases, Generated by QSAR and Computational Design. J. Enzyme Inhib. Med. Chem..

[55] Mishra C.B., Tiwari M., Supuran C.T. (2020). Progress in the Development of Human Carbonic Anhydrase Inhibitors and Their Pharmacological Applications: Where Are We Today?. Med. Res. Rev..

[56] Ciccone L., Cerri C., Nencetti S., Orlandini E. (2021). Carbonic Anhydrase Inhibitors and Epilepsy: State of the Art and Future Perspectives. Molecules.

[57] Kida E., Palminiello S., Golabek A.A., Walus M., Wierzba-Bobrowicz T., Rabe A., Albertini G., Wisniewski K.E. (2006). Carbonic Anhydrase II in the Developing and Adult Human Brain. J. Neuropathol. Exp. Neurol..

[58] Aspatwar A., Tolvanen M.E.E., Ortutay C., Parkkila S. (2014). Carbonic Anhydrase Related Proteins: Molecular Biology and Evolution. Subcell. Biochem..

[59] Mishra C.B., Kumari S., Angeli A., Bua S., Tiwari M., Supuran C.T. (2018). Discovery of Benzenesulfonamide Derivatives as Carbonic Anhydrase Inhibitors with Effective Anticonvulsant Action: Design, Synthesis, and Pharmacological Evaluation. J. Med. Chem..

[60] Balestri F., Moschini R., Mura U., Cappiello M., Corso A.D. (2022). In Search of Differential Inhibitors of Aldose Reductase. Biomolecules.

[61] Veeresham C., Rama Rao A., Asres K. (2014). Aldose Reductase Inhibitors of Plant Origin. Phytother. Res. PTR.

[62] Grewal A.S., Bhardwaj S., Pandita D., Lather V., Sekhon B.S. (2016). Updates on Aldose Reductase Inhibitors for Management of Diabetic Complications and Non-Diabetic Diseases. Mini Rev. Med. Chem..

[63] Huang Y.-K., Liu C.-C., Wang S., Cheng H.-C., Meadows C., Chang K.-C. (2022). The Role of Aldose Reductase in Beta-Amyloid-Induced Microglia Activation. Int. J. Mol. Sci..

[64] SIB Swiss Institute of Bioinformatics | Expasy. https://www.expasy.org/.

[65] Lipinski C.A., Lombardo F., Dominy B.W., Feeney P.J. (2001). Experimental and Computational Approaches to Estimate Solubility and Permeability in Drug Discovery and Development Settings. Adv. Drug Deliv. Rev..

[66] Veber D.F., Johnson S.R., Cheng H.-Y., Smith B.R., Ward K.W., Kopple K.D. (2002). Molecular Properties That Influence the Oral Bioavailability of Drug Candidates. J. Med. Chem..

[67] Egan W.J., Merz K.M., Baldwin J.J. (2000). Prediction of Drug Absorption Using Multivariate Statistics. J. Med. Chem..

[68] Daina A., Michielin O., Zoete V. (2017). SwissADME: A Free Web Tool to Evaluate Pharmacokinetics, Drug-Likeness and Medicinal Chemistry Friendliness of Small Molecules. Sci. Rep..

[69] Martin Y.C. (2005). A Bioavailability Score. J. Med. Chem..

[70] Kesarwani K., Gupta R. (2013). Bioavailability Enhancers of Herbal Origin: An Overview. Asian Pac. J. Trop. Biomed..

[71] Nistorescu S., Gradisteanu Pircalabioru G., Udrea A.-M., Simon A., Pascu M.L., Chifiriuc M.-C. (2020). Laser-Irradiated Chlorpromazine as a Potent Anti-Biofilm Agent for Coating of Biomedical Devices. Coatings.

[72] Udrea A.-M., Dinache A., Pagès J.-M., Pirvulescu R.A. (2021). Quinazoline Derivatives Designed as Efflux Pump Inhibitors: Molecular Modeling and Spectroscopic Studies. Molecules.

[73] Pervin M., Unno K., Takagaki A., Isemura M., Nakamura Y. (2019). Function of Green Tea Catechins in the Brain: Epigallocatechin Gallate and Its Metabolites. Int. J. Mol. Sci..

[74] Supuran C.T. (2020). Coumarin Carbonic Anhydrase Inhibitors from Natural Sources. J. Enzyme Inhib. Med. Chem..

[75] Bickerton G.R., Paolini G.V., Besnard J., Muresan S., Hopkins A.L. (2012). Quantifying the Chemical Beauty of Drugs. Nat. Chem..

[76] Ghose A.K., Viswanadhan V.N., Wendoloski J.J. (1999). A Knowledge-Based Approach in Designing Combinatorial or Medicinal Chemistry Libraries for Drug Discovery. 1. A Qualitative and Quantitative Characterization of Known Drug Databases. J. Comb. Chem..

[77] Avram S., Mernea M., Borcan F., Mihailescu D. (2016). Evaluation of the Therapeutic Properties of Mastoparan- and Sifuvirtide- Derivative Antimicrobial Peptides Using Chemical Structure-Function Relationship—in Vivo and in Silico Approaches. Curr. Drug Deliv..

[78] Daina A., Zoete V. (2016). A BOILED-Egg To Predict Gastrointestinal Absorption and Brain Penetration of Small Molecules. ChemMedChem.

[79] Moret M., Pachon Angona I., Cotos L., Yan S., Atz K., Brunner C., Baumgartner M., Grisoni F., Schneider G. (2023). Leveraging Molecular Structure and Bioactivity with Chemical Language Models for de Novo Drug Design. Nat. Commun..

[80] Dumitrascu F., Udrea A.-M., Caira M.R., Nuta D.C., Limban C., Chifiriuc M.C., Popa M., Bleotu C., Hanganu A., Dumitrescu D. (2022). In Silico and Experimental Investigation of the Biological Potential of Some Recently Developed Carprofen Derivatives. Molecules.

[81] Putz M.V., Duda-Seiman C., Duda-Seiman D., Putz A.-M., Alexandrescu I., Mernea M., Avram S. (2016). Chemical Structure-Biological Activity Models for Pharmacophores’ 3D-Interactions. Int. J. Mol. Sci..

[82] Caron G., Digiesi V., Solaro S., Ermondi G. (2020). Flexibility in Early Drug Discovery: Focus on the beyond-Rule-of-5 Chemical Space. Drug Discov. Today.

[83] Raevsky O.A., Grigorev V.Y., Polianczyk D.E., Raevskaja O.E., Dearden J.C. (2019). Aqueous Drug Solubility: What Do We Measure, Calculate and QSPR Predict?. Mini Rev. Med. Chem..

[84] Vraka C., Mijailovic S., Fröhlich V., Zeilinger M., Klebermass E.-M., Wadsak W., Wagner K.-H., Hacker M., Mitterhauser M. (2018). Expanding LogP: Present Possibilities. Nucl. Med. Biol..

[85] Chomicki D., Kharchenko O., Skowronski L., Kowalonek J., Kozanecka-Szmigiel A., Szmigiel D., Smokal V., Krupka O., Derkowska-Zielinska B. (2020). Physico-Chemical and Light-Induced Properties of Quinoline Azo-Dyes Polymers. Int. J. Mol. Sci..

[86] Pires D.E.V., Blundell T.L., Ascher D.B. (2015). PkCSM: Predicting Small-Molecule Pharmacokinetic and Toxicity Properties Using Graph-Based Signatures. J. Med. Chem..

[87] Udrea A.M., Gradisteanu Pircalabioru G., Boboc A.A., Mares C., Dinache A., Mernea M., Avram S. (2021). Advanced Bioinformatics Tools in the Pharmacokinetic Profiles of Natural and Synthetic Compounds with Anti-Diabetic Activity. Biomolecules.

[88] Avram S., Udrea A.M., Negrea A., Ciopec M., Duteanu N., Postolache C., Duda-Seiman C., Duda-Seiman D., Shaposhnikov S. (2019). Prevention of Deficit in Neuropsychiatric Disorders through Monitoring of Arsenic and Its Derivatives as Well as Through Bioinformatics and Cheminformatics. Int. J. Mol. Sci..

[89] Avram S., Mernea M., Mihailescu D., Duda-Seiman D., Duda-Seiman C. (2013). Advanced QSAR Methods Evaluated Polycyclic Aromatic Compounds Duality as Drugs and Inductors in Psychiatric Disorders. Curr. Org. Chem..

[90] Hikino H., Konno C., Takata H., Yamada Y., Yamada C., Ohizumi Y., Sugio K., Fujimura H. (1980). Antiinflammatory Principles of Aconitum Roots. J. Pharmacobiodyn..

[91] Bai L., Li X., He L., Zheng Y., Lu H., Li J., Zhong L., Tong R., Jiang Z., Shi J. (2019). Antidiabetic Potential of Flavonoids from Traditional Chinese Medicine: A Review. Am. J. Chin. Med..

[92] Daina A., Michielin O., Zoete V. (2019). SwissTargetPrediction: Updated Data and New Features for Efficient Prediction of Protein Targets of Small Molecules. Nucleic Acids Res..

[93] Negrea E., Oancea P., Leonties A., Ana Maria U., Avram S., Raducan A. (2023). Spectroscopic Studies on Binding of Ibuprofen and Drotaverine with Bovine Serum Albumin. J. Photochem. Photobiol. Chem..

[94] Molecular Operating Environment (MOE) (2022). 2022.02 Chemical Computing Group ULC, 1010 Sherbooke St. West, Suite #910, Montreal, QC, Canada, H3A 2R7. https://www.chemcomp.com/Research-Citing_MOE.htm.

[95] Open Babel: An Open Chemical Toolbox | Journal of Cheminformatics | Full Text. https://jcheminf.biomedcentral.com/articles/10.1186/1758-2946-3-33.

[96] Harrison D.H., Bohren K.M., Ringe D., Petsko G.A., Gabbay K.H. (1994). An Anion Binding Site in Human Aldose Reductase: Mechanistic Implications for the Binding of Citrate, Cacodylate, and Glucose 6-Phosphate. Biochemistry.

[97] Morris G.M., Huey R., Olson A.J. (2008). Using AutoDock for Ligand-Receptor Docking. Curr. Protoc. Bioinforma..

[98] Gao X., Zhang X., Hu J., Xu X., Zuo Y., Wang Y., Ding J., Xu H., Zhu S. (2018). Aconitine Induces Apoptosis in H9c2 Cardiac Cells via Mitochondria-mediated Pathway. Mol. Med. Rep..

[99] Ravindran J., Gupta N., Agrawal M., Bala Bhaskar A.S., Lakshmana Rao P.V. (2011). Modulation of ROS/MAPK Signaling Pathways by Okadaic Acid Leads to Cell Death via, Mitochondrial Mediated Caspase-Dependent Mechanism. Apoptosis Int. J. Program. Cell Death.

[100] Anjum F., Ali F., Mohammad T., Shafie A., Akhtar O., Abdullaev B., Hassan I. (2021). Discovery of Natural Compounds as Potential Inhibitors of Human Carbonic Anhydrase II: An Integrated Virtual Screening, Docking, and Molecular Dynamics Simulation Study. Omics J. Integr. Biol..

[101] Taslimi P., Caglayan C., Gulcin İ. (2017). The Impact of Some Natural Phenolic Compounds on Carbonic Anhydrase, Acetylcholinesterase, Butyrylcholinesterase, and α-Glycosidase Enzymes: An Antidiabetic, Anticholinergic, and Antiepileptic Study. J. Biochem. Mol. Toxicol..

[102] Aggul A.G., Uzun N., Kuzu M., Taslimi P., Gulcin I. (2022). Some Phenolic Natural Compounds as Carbonic Anhydrase Inhibitors: An in Vitro and in Silico Study. Arch. Pharm. (Weinheim).

[103] Sethi K.K., Sahoo S.K., Pichikala J.N., Suresh P. (2012). Carbonic Anhydrase I and II Inhibition with Natural Products: Caffeine and Piperine. J. Enzyme Inhib. Med. Chem..

[104] BIOVIA (2021). Dassault Systèmes, [Discovery Studio Visualizer], [V21.1.0.20298].

[105] Chahal V., Kakkar R. (2022). A Combination Strategy of Structure-Based Virtual Screening, MM-GBSA, Cross Docking, Molecular Dynamics and Metadynamics Simulations Used to Investigate Natural Compounds as Potent and Specific Inhibitors of Tumor Linked Human Carbonic Anhydrase IX. J. Biomol. Struct. Dyn..

